# Cognitive impairment and associated factors in elderly patients with schizophrenia: a retrospective observational study with phenotype analysis

**DOI:** 10.3389/fpsyt.2026.1789211

**Published:** 2026-04-30

**Authors:** Yanping Cai, Yinxia Wu, Danjing Yao, Ying Xu

**Affiliations:** Department of Geriatric Psychiatry, Inpatient Area VII, Huzhou Third Municipal Hospital, the Affiliated Hospital of Huzhou University, Huzhou, Zhejiang, China

**Keywords:** cognitive impairment, elderly schizophrenia, influencing factors, nursing management, social support

## Abstract

**Objective:**

To examine the prevalence of cognitive impairment and factors associated with cognitive impairment among elderly patients with schizophrenia, and to explore potential clinical phenotypes using a clustering approach.

**Methods:**

A retrospective cross-sectional study was conducted among 149 elderly inpatients with schizophrenia. Cognitive impairment was defined as Montreal Cognitive Assessment (MoCA) score < 26. Clinical characteristics, metabolic indicators, activities of daily living (ADL), and perceived social support (MSPSS) were collected. Multivariate logistic regression was performed to identify factors associated with cognitive impairment. Model classification performance was evaluated using a 2 × 2 table. K-means clustering was applied to explore cognitive and social functioning phenotypes.

**Results:**

The prevalence of cognitive impairment among elderly patients with schizophrenia was 47.7%. Multivariate logistic regression analysis showed that higher MSPSS total score (OR = 0.609, 95% CI: 0.469-0.791, p < 0.001) and higher ADL score (OR = 0.553, 95% CI: 0.379-0.806, p = 0.002) were independently associated with lower odds of cognitive impairment. Diabetes mellitus was associated with increased odds (OR = 7.735, 95% CI: 1.129-52.979, p = 0.037), although the wide confidence interval indicates limited precision. The model demonstrated good classification performance (accuracy = 96.6%). K-means clustering identified three phenotypes (optimal functioning, mild-to-moderate impairment, and severe impairment), which were significantly associated with cognitive impairment status (χ² = 137.58, p < 0.001).

**Conclusion:**

Cognitive impairment is prevalent among elderly patients with schizophrenia and is associated with reduced social support, lower functional independence, and diabetes. Cluster analysis suggests heterogeneity in cognitive and psychosocial profiles, supporting the potential value of stratified nursing management. Prospective studies are warranted to validate these findings.

## 1 Introduction

Schizophrenia is a severe psychiatric disorder characterized by early onset, chronic course, high relapse rate, and substantial disability (1). With the acceleration of population aging, the number of elderly patients with schizophrenia is increasing annually, and their long-term disease burden and care needs are becoming increasingly prominent (2). Compared with younger patients, elderly patients with schizophrenia not only contend with persistent effects of schizophrenia but are also more susceptible to multiple chronic comorbidities and functional decline (3), making them a particularly vulnerable population in the field of mental health.

Cognitive impairment is one of the core deficits of schizophrenia, commonly manifesting as impairments in attention, memory, executive function, and social cognition. These impairments exert profound effects on treatment adherence, social functioning, and quality of life. Cognitive impairment in elderly patients with schizophrenia is often more severe than that observed in younger patients, driven not only by the illness itself but also by factors including brain aging, degenerative changes in neurotransmitter systems, chronic comorbidities, and long-term medication use. Consequently, cognitive impairment in this population tends to be persistent, with relatively slow recovery, which makes interventions challenging (4, 5).

Cognitive impairment among elderly patients with schizophrenia exhibits considerable heterogeneity and influenced by multiple factors. Research indicates that the global lifetime prevalence of schizophrenia is approximately 1%, with no significant sex differences. However, elderly patients are often affected by comorbid conditions such as hypertension, coronary heart disease, and diabetes mellitus, which may exacerbate cognitive impairment through mechanisms such as metabolic disturbances or vascular damage (6). In addition, psychosocial factors, including educational level, lifestyle, and social support, also play critical roles in maintaining cognitive function (7).

From a nursing perspective, cognitive impairment substantially increases the complexity of care. Patients often demonstrate poor medication adherence, reduced self-care ability, and heightened risk of accidents, which impose a heavy burden on families and society (8). Therefore, assessing the prevalence and key determinants of cognitive impairment in elderly patients with schizophrenia is essential for developing individualized nursing intervention strategies, delaying functional decline, and improving rehabilitation outcomes.

At present, most studies focus on younger and middle-aged patients, while systematic research targeting elderly patients remains limited. Evidence-based guidance for nursing interventions in this population is lacking. Future nursing interventions should not only emphasize disease management but also integrate cognitive rehabilitation, chronic disease management, and social support, thereby exploring multidimensional and comprehensive care models. Such research will contribute to improving the prognosis and quality of life of elderly patients with schizophrenia and provide new directions for psychiatric nursing practice.

## 2 Materials and methods

### 2.1 Study population

This retrospective cohort study included 149 elderly patients with schizophrenia who were admitted to the Department of Psychiatry of our hospital between October 2023 and September 2024. Based on previous literature (9) and the expected effect size (ES = 0.30), the minimum sample size was set at 120 cases to ensure the statistical power (power = 0.8, α = 0.05) for analyzing the primary outcome (prevalence of cognitive impairment). Ultimately, 149 patients were enrolled, satisfying the requirements for statistical analysis.

The study adhered to the principles of the Declaration of Helsinki and was approved by the Ethics Committee of Huzhou Third Municipal Hospital, the Affiliated Hospital of Huzhou University (Approval No. 2025-248). All data were anonymized to protect participant privacy. The study was conducted in a tertiary psychiatric hospital in Zhejiang Province, China, which includes specialized geriatric psychiatry inpatient units. All participants were recruited during inpatient hospitalization in a clinically stable phase. Cognitive and psychosocial assessments were performed during hospitalization. The average length of hospital stay was approximately 30 days. The requirement for written informed consent was waived by the Ethics Committee due to the retrospective nature of the study and the use of anonymized data.

The inclusion criteria were as follows: (i) age ≥60 years; (ii) diagnosis of schizophrenia according to the Diagnostic and Statistical Manual of Mental Disorders, Fifth Edition (DSM-5) (10); (iii) disease duration ≥1 year and clinically stable condition at enrollment, with continuous and regular oral administration of antipsychotic medications for > 1 year during the maintenance phase, and ability to complete cognitive function assessments.

The exclusion criteria were: (i) comorbid severe neurological disorders (e.g., stroke, Alzheimer’s disease, Parkinson’s disease) or progressive cognitive impairment; (ii) severe hearing or visual impairment interfering with cognitive assessment; (iii) recent acute psychotic episode or history of substance abuse; (iv) major physical illness or surgical history likely to interfere with cognitive function assessment; (v) alcohol dependence or psychoactive substance dependence; (vi) electroconvulsive therapy within the past 3 months.

### 2.2 Data collection

All data were retrospectively collected from the hospital’s electronic medical record system by two trained researchers and cross-checked by double entry to ensure accuracy and completeness. The collected data included: Demographic characteristics: age, sex, body mass index (BMI), and educational level. Disease-related variables: disease duration, number of relapses, and family history of psychiatric disorders. Physical comorbidities: presence of hypertension or type 2 diabetes mellitus (T2DM). Cognitive function assessment: conducted using the Montreal Cognitive Assessment (MoCA) (11), administered face-to-face by psychiatrists trained in a standardized protocol. The MoCA has a total score of 30, with a score <26 indicating cognitive impairment. For patients with ≤12 years of education, one additional point was added to the total score before determination. Laboratory indicators: glycosylated hemoglobin (HbA1c), total cholesterol (TC), low-density lipoprotein cholesterol (LDL-C), high-density lipoprotein cholesterol (HDL-C), triglycerides (TG), and cystatin C (CysC). Psychosocial factors: during the stable phase of the illness, the Multidimensional Scale of Perceived Social Support (MSPSS) (12) was administered face-to-face by trained researchers. The MSPSS includes three dimensions, support from family, friends, and significant others, with higher scores indicating better perceived social support. Activities of daily living (ADL): assessed using the Barthel Index (13) by trained researchers, covering activities such as eating, bathing, dressing, toileting, and mobility. The total score ranges from 0 to 100, with higher scores indicating better self-care ability in daily life. All assessors received standardized training prior to data collection to ensure consistency. Given the retrospective nature of the study, blinding to clinical information was not feasible.

### 2.3 Statistical analysis

All data were analyzed using IBM SPSS Statistics version 26.0 (IBM Corp., Armonk, NY, USA). Measurement data with a normal distribution were expressed as mean ± standard deviation ( $\bar{x}$ ± s), and comparisons between groups were performed using the independent-samples t test. Measurement data not following a normal distribution were expressed as median (interquartile range) [M (P25, P75)], and comparisons between groups were performed using the Mann–Whitney U test. Categorical data were expressed as numbers and percentages [n (%)], and comparisons between groups were performed using the χ² test or Fisher’s exact test. Multivariate logistic regression analysis was used to identify independent risk factors for cognitive impairment, with results presented as odds ratios (ORs) and 95% confidence intervals (CIs). The Hosmer–Lemeshow test was used to evaluate the goodness-of-fit of the model. Receiver operating characteristic (ROC) curves were plotted, and the area under the curve (AUC) was calculated to assess the predictive performance of the model. All tests were two-tailed, and *p* < 0.05 was considered statistically significant. Effect sizes were reported as Pearson’s r (point-biserial correlation for continuous variables and phi coefficient for dichotomous variables).

In addition, K-means clustering analysis was performed to identify data-driven patterns across cognitive, functional, and psychosocial variables. K-means is an unsupervised clustering algorithm that partitions observations into k clusters by minimizing within-cluster variance. The optimal number of clusters was determined using the elbow method, considering within-cluster sum of squares and interpretability of the resulting clusters. Solutions with k = 2, 3, and 4 were examined, and a three-cluster solution was selected as it provided the clearest separation of cognitive and psychosocial profiles while maintaining interpretability and avoiding over-fragmentation observed in the four-cluster solution.

## 3 Results

### 3.1 Clinical characteristics of patients

A total of 149 elderly patients with schizophrenia were included. The mean age was 73.81 ± 4.07 years (range 63–83 years), with 54.4% female and 45.6% male. The mean years of education were 9.95 ± 3.49 years, and the mean disease duration was 20.17 ± 6.95 years. The median number of relapses was 3.0 (interquartile range, 1.0–4.0). The mean BMI was 24.17 ± 2.85 kg/m². The mean ADL score was 86.38 ± 6.21. The mean MoCA score was 23.13 ± 6.58. The mean MSPSS score was 51.42 ± 10.39.

Comorbidity profiles revealed that 61.7% of patients had hypertension, 33.6% had diabetes mellitus, 15.4% had chronic kidney disease, and 16.1% had a family history of psychiatric disorders. Laboratory findings were as follows: HbA1c 6.15 ± 0.72%, TC 4.97 ± 0.74 mmol/L, LDL-C 2.74 ± 0.62 mmol/L, HDL-C 1.26 ± 0.33 mmol/L, TG 1.50 ± 0.52 mmol/L, and CysC 1.10 ± 0.24 mg/L.

### 3.2 Prevalence of cognitive impairment

**Figure 1:**
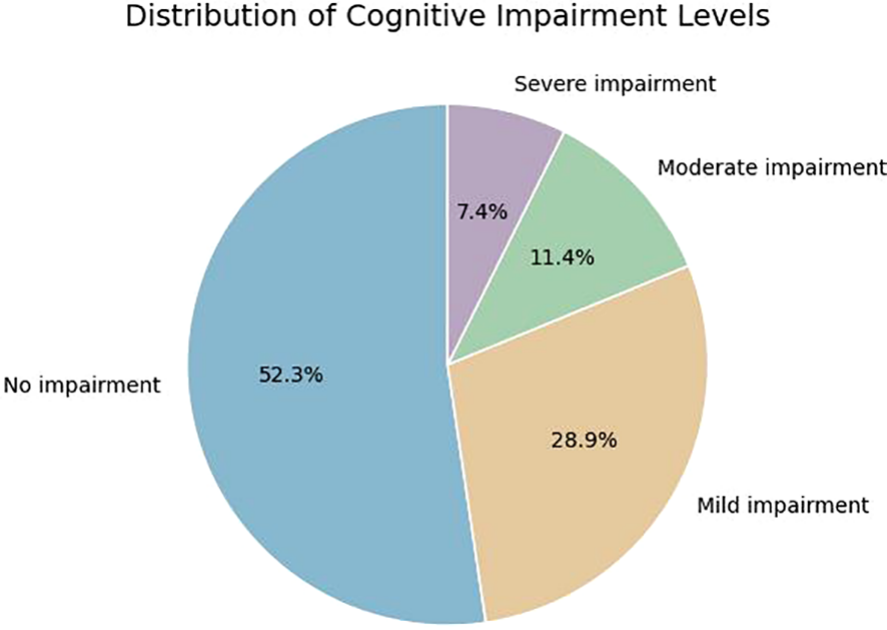
Prevalence of cognitive impairment.

Based on MoCA assessment, 71 out of 149 patients (47.65%) met criteria for cognitive impairment. Among these, 78 patients had no impairment, 43 had mild impairment, 17 had moderate impairment, and 11 had severe impairment (**Figure 1**).

### 3.3 Comparison of clinical characteristics between patients with and without cognitive impairment

Significant differences were observed between the cognitive impairment and non-impairment groups in terms of age, years of education, disease duration, number of relapses, ADL score, MoCA total score, MSPSS total score, HbA1c, hypertension, diabetes mellitus, and CysC levels (*p* < 0.05). No significant differences were found for sex, chronic kidney disease, family history of psychiatric disorders, BMI, or lipid parameters (TC, LDL-C, HDL-C, TG) (*p* > 0.05). Although mean BMI values approached the threshold between normal and overweight categories, BMI category distributions were comparable between groups (*p* > 0.05) (**Table 1**).

**Table 1:** Comparison of clinical characteristics between elderly patients with schizophrenia with and without cognitive impairment.

Variables	Non-impairment groups (n=78)	Cognitive impairment group (n=71)	*χ²/t*	*p*	*r*
Sex n (%)			0.001	0.974	0.039
Female	43 (55.1%)	38 (53.5%)			
Male	35 (44.9%)	33 (46.5%)			
Hypertension n (%)			10.631	0.001	0.267
No	40 (51.3%)	17 (23.9%)			
Yes	38 (48.7%)	54 (76.1%)			
Diabetes mellitus n (%)			7.107	0.008	0.218
No	60 (76.9%)	39 (54.9%)			
Yes	18 (23.1%)	32 (45.1%)			
Chronic kidney disease n (%)			1.247	0.264	0.091
No	63 (80.8%)	63 (88.7%)			
Yes	15 (19.2%)	8 (11.3%)			
Family history of psychiatric disorders n (%)			0.225	0.635	0.039
No	67 (85.9%)	58 (81.7%)			
Yes	11 (14.1%)	13 (18.3%)			
Age (years)	72.86 ± 4.26	74.85 ± 3.60	-3.081	0.002	0.244
Years of education	11.38 ± 3.14	8.38 ± 3.19	5.790	<0.001	-0.431
Disease duration (years)	18.34 ± 6.87	22.18 ± 6.51	-3.499	0.001	0.277
Number of relapses	1.79 ± 1.32	3.96 ± 1.74	-8.600	<0.001	0.579
BMI (kg/m²)	24.11 ± 2.64	24.24 ± 3.08	-0.285	0.776	0.024
BMI category n (%)			3.169	0.205	0.146
Normal (<25)	51 (65.4%)	39 (54.9%)			
Overweight (25–29.9)	24 (30.8%)	31 (43.7%)			
Obese (≥30)	3 (3.8%)	1 (1.4%)			
ADL score	90.18 ± 5.53	82.21 ± 3.76	10.177	<0.001	-0.643
MoCA total score	27.91 ± 1.40	17.89 ± 6.01	14.312	<0.001	-0.763
MSPSS total score	58.94 ± 6.89	43.17 ± 6.64	14.197	<0.001	-0.760
HbA1c(%)	5.86 ± 0.57	6.47 ± 0.73	-5.731	<0.001	0.427
TC(mmol/L)	4.94 ± 0.75	5.01 ± 0.73	-0.546	0.586	0.045
LDL-C(mmol/L)	2.66 ± 0.60	2.82 ± 0.64	-1.538	0.126	0.126
HDL-C (mmol/L)	1.26 ± 0.33	1.26 ± 0.33	-0.154	0.878	0.013
TG(mmol/L)	1.53 ± 0.53	1.47 ± 0.52	0.657	0.512	-0.054
CysC(mg/L)	0.99 ± 0.20	1.22 ± 0.21	-6.776	<0.001	0.488

### 3.4 Multivariate logistic regression analysis of factors associated with cognitive impairment in elderly patients with schizophrenia

To examine factors associated with cognitive impairment, multivariate logistic regression analysis was performed with cognitive impairment (0 = no, 1 = yes) as the dependent variable. Variables showing significant differences in univariate analyses were preliminarily entered into the model. To ensure model stability and minimize overfitting given the number of outcome events (n = 71), a parsimonious modeling strategy was adopted, guided by multicollinearity diagnostics and events-per-variable considerations. MoCA total score was excluded as it defines cognitive status. Number of relapses showed high collinearity with disease duration (Spearman’s r = 0.72; variance inflation factor > 5) and was excluded. HbA1c was highly correlated with diabetes mellitus status (r = 0.68); therefore, diabetes mellitus was retained as the clinically more interpretable variable, and HbA1c was excluded to reduce redundancy. CysC demonstrated substantial collinearity with chronic kidney disease and ADL score, and its inclusion in the multivariable model resulted in inflated standard errors and marked fluctuation in coefficient estimates across alternative specifications, indicating instability. Accordingly, CysC was excluded to improve model robustness.

The final multivariable model included age, years of education, disease duration, ADL score, MSPSS total score, hypertension, and diabetes mellitus. As shown in **Table 2**, higher MSPSS total score (OR = 0.609, 95% CI: 0.469–0.791, p < 0.001) and higher ADL score (OR = 0.553, 95% CI: 0.379–0.806, p = 0.002) were independently associated with lower odds of cognitive impairment. Diabetes mellitus was associated with increased odds of cognitive impairment (OR = 7.735, 95% CI: 1.129–52.979, p = 0.037), though the wide CI suggests limited precision and warrants cautious interpretation. Age, education, disease duration, and hypertension were not independently significant after adjustment.

**Table 2:** Multivariate logistic regression analysis of factors associated with cognitive impairment in elderly patients with schizophrenia.

Variable	β	SE	OR	95% CI	p
Age	0.03	0.087	1.031	0.870–1.229	0.725
Years of education	-0.169	0.110	0.845	0.679–1.051	0.131
Disease duration	0.034	0.051	1.035	0.938–1.144	0.506
ADL score	-0.593	0.194	0.553	0.379–0.806	0.002
MSPSS total score	-0.496	0.134	0.609	0.469–0.791	<0.001
Hypertension	0.686	0.473	1.986	0.786–5.017	0.147
Diabetes mellitus	2.045	0.982	7.735	1.129–52.979	0.037

OR, odds ratio; CI, confidence interval; SE, standard error.

The classification table (**Table 3**) showed that 75 of 78 non-impaired patients and 69 of 71 impaired patients were correctly classified. The overall classification accuracy was 0.966, with a sensitivity of 0.972 and specificity of 0.962. Model performance was evaluated using the development dataset only; no internal cross-validation or external validation was conducted. Therefore, predictive performance estimates may be optimistic.

**Table 3:** 2×2 classification table.

	Predicted non-impaired	Predicted impaired
Observed Non-impaired	75	3
Observed Impaired	2	69

### 3.5 Cognitive and social functioning phenotypes

**Figure 2:**
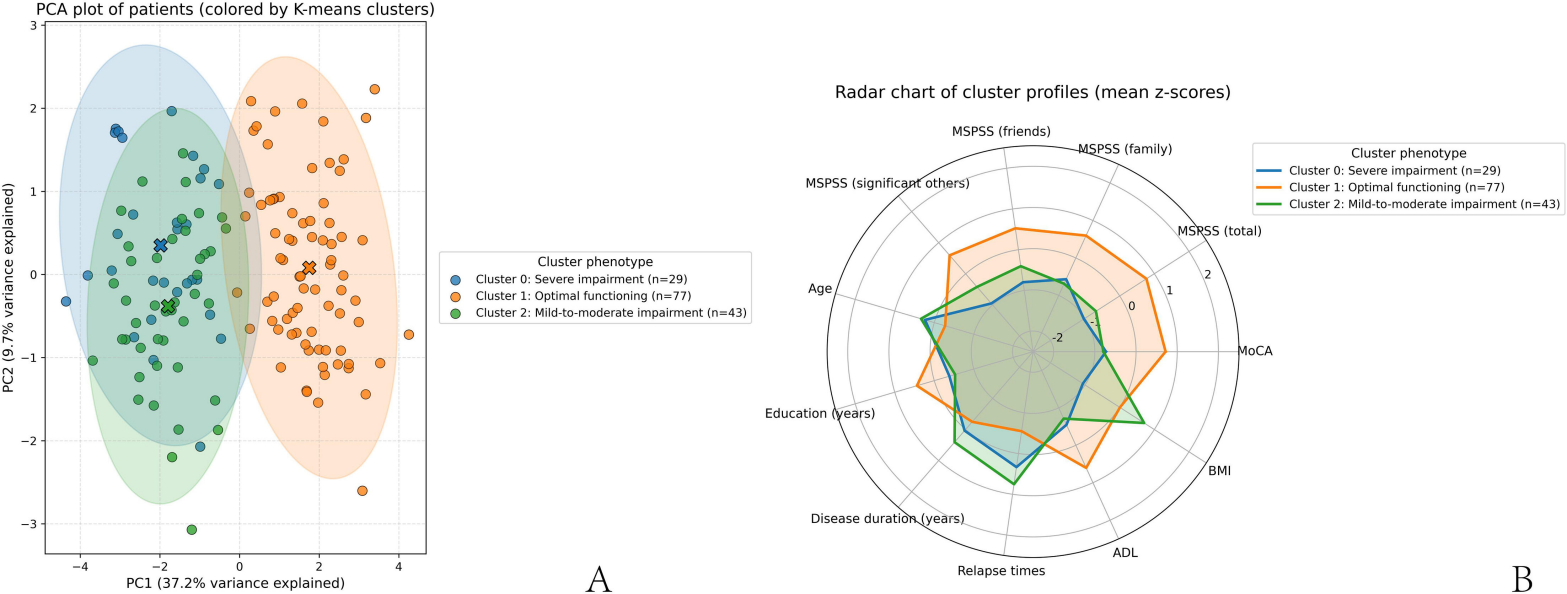
K-means cluster analysis of cognitive and social function phenotypes in patients. **(A)** PCA two-dimensional score plot; **(B)** Radar chart of standardized (z-score) mean values across clusters.

To explore potential phenotypic differences in cognitive function, social support, and disease characteristics, a K-means clustering analysis was performed using MoCA total score, MSPSS total and subdimension scores, age, years of education, disease duration, number of relapses, ADL score, and BMI. Prior to clustering, principal component analysis (PCA) was applied for dimensionality reduction and visualization of sample distribution. All variables were standardized (z-scores) prior to PCA to eliminate scale effects. PCA was used solely for visualization and exploratory interpretation of the data structure; clustering was performed on the standardized original variables rather than on principal component scores. PCA results showed that the first principal component (PC1) explained 37.19% of the variance, and the second principal component (PC2) explained 9.70%, with the first two components accounting for a cumulative variance of 46.88%. A two-dimensional scatter plot showed clear separation between clusters (**Figure 2A**). Factor loadings for PC1 and PC2 are presented in **Supplementary Table 1**. PC1 was primarily driven by high positive loadings of MoCA, ADL, and MSPSS scores and negative loadings of disease duration and relapse frequency, suggesting that this component reflects a general cognitive–functional axis. PC2 showed relatively higher loadings for age and BMI, suggesting that this component may reflect a demographic or physical status-related dimension; however, given the modest proportion of explained variance, this interpretation should be considered exploratory.

Based on the predefined three-cluster solution, K-means clustering identified three groups: Cluster 1 (n = 77), Cluster 2 (n = 43), and Cluster 0 (n = 29). Radar charts illustrated the mean values of indicators across clusters (**Figure 2B**). For visualization purposes, variables displayed in the radar chart were standardized (z-scores) to allow meaningful cross-variable comparison across different scales. (i) Cluster 1 showed the highest levels of cognitive function, social support, and ADL, along with shorter disease duration, fewer relapses, and longer educational history, representing an optimal functioning pattern. (ii) Cluster 2 demonstrated the lowest cognitive function and social support, longest disease duration, highest relapse frequency, and lowest ADL scores, representing a severe impairment pattern. (iii) Cluster 0 displayed intermediate cognitive function and disease characteristics, with relatively lower social support than Cluster 1, representing a mild-to-moderate impairment phenotype.

No significant differences were observed in BMI and age across clusters, suggesting a limited contribution of these variables to cluster differentiation. PCA and radar visualization provided preliminary insights into heterogeneity within the sample (**Table 4**). Scree plot of eigenvalues is presented in **Supplementary Figure 1**.

**Table 4:** Interpretation of clinical phenotypes.

Cluster	Characteristics	Clinical interpretation
Mild-to-moderate impairment (Cluster 0)	Average cognition, low social support	Mild-to-moderate cognitive impairment; cognitive training and social support interventions are recommended
Optimal function (Cluster 1)	Good cognition, high social support, short disease duration	Best overall clinical function; focus on maintaining function and providing cognitive training
Severe impairment (Cluster 2)	Poor cognition, low social support, long disease duration with frequent relapses	Severe cognitive impairment; requires comprehensive chronic disease management and intensive nursing care

Cross-tabulation analysis further examined the relationship between cluster membership and the original two-group classification (cognitively impaired *vs*. non-impaired). As shown in **Table 5**, Cluster 2 consisted predominantly of cognitively impaired participants (43/43), whereas Cluster 1 included mainly non-impaired participants (76/77). Cluster 0 showed a mixed distribution (27 impaired *vs*. 2 non-impaired). The association between cluster membership and cognitive impairment status was statistically significant (χ² = 137.58, p < 0.001) (**Table 5**).

**Table 5:** Cross-classification of cluster membership and cognitive impairment status.

Cluster	Non-impaired (0)	Impaired (1)	Total
Cluster 1 (n=77)	76	1	77
Cluster 2 (n=43)	0	43	43
Cluster 0 (n=29)	2	27	29
Total	78	71	149

## 4 Discussion

### 4.1 Prevalence of cognitive impairment and associated clinical characteristics

This study found that 47.7% of elderly participants with schizophrenia met the screening criteria for cognitive impairment (defined by MoCA < 26), indicating a substantial cognitive burden in this population. Although the MoCA is a screening tool rather than a diagnostic instrument, the findings suggest a high burden of cognitive impairment in this clinical sample.Previous epidemiological studies have reported that the prevalence of cognitive impairment among community-dwelling older adults typically ranges from approximately 10% to 25%, depending on age structure and assessment tools used (14). In contrast, studies focusing on older adults with schizophrenia have documented substantially higher prevalence rates, often ranging between 40% and 60% (15). Therefore, the 47.7% prevalence observed in our sample appears consistent with the elevated cognitive vulnerability reported in aging schizophrenia populations, although differences in sampling methods and diagnostic criteria should be considered. It should be noted that applying the conventional cutoff (<26) in an elderly psychiatric population with relatively low educational attainment may have resulted in an overestimation of impairment, despite the +1 education correction. Cognitive impairment was associated with longer disease duration and more frequent relapses. While the present study cannot clarify underlying mechanisms, prior neurobiological research implicates dysfunction within prefrontal-hippocampal circuits in the context of persistent symptoms and chronic stress exposure (16). Although such mechanisms were not directly examined in the current analysis, they may provide a broader theoretical context for understanding the observed clinical associations. Lower educational attainment was also more common among cognitively impaired patients, consistent with the cognitive reserve hypothesis, which posits that reduced educational exposure may limit neural resilience to neuropathological processes. However, lower educational attainment may also reflect lifelong differences in baseline cognitive capacity rather than disease-related cognitive decline. Individuals with fewer years of formal education may have had lower premorbid cognitive resources, which could increase vulnerability to later-life cognitive impairment independent of schizophrenia progression or aging effects. Therefore, the observed association between education and cognitive impairment may represent pre-existing variation rather than disease-specific deterioration. Longitudinal studies incorporating premorbid cognitive estimates are needed to clarify this relationship. Furthermore, patients with cognitive impairment had significantly lower ADL and MSPSS scores, indicating a close association between cognitive status, functional ability, and perceived social support. However, given the cross-sectional design, the directionality of these relationships cannot be determined. Reduced social support and functional capacity may represent contributing factors, consequences, or parallel manifestations of cognitive impairment. It is also plausible that cognitive impairment may contribute to social withdrawal, communication difficulties, and reduced participation in family and community activities, thereby diminishing social network size and perceived support. Thus, the observed associations may reflect reciprocal or bidirectional processes rather than unidirectional effects. These findings are consistent with the “dual decline” theory of cognition and function (17).

### 4.2 Interpretation of factors associated with cognitive impairment

Multivariate logistic regression analysis indicated that higher MSPSS and ADL scores were independently associated with lower odds of cognitive impairment. Social support has been linked in previous cohort studies to better cognitive outcomes in older adults (18), and cognitive deficits have been strongly associated with everyday functional disability in schizophrenia populations (19), highlighting the close interrelationship between cognitive and functional domains. Similarly, higher ADL scores were independently protective. Functional independence reflects daily engagement and environmental interaction, both of which have been correlated with cognitive performance (20). Because MSPSS and ADL were continuous variables, the reported odds ratios represent the change in odds per 1-point increase. For improved interpretability, a 5-point increase in MSPSS corresponds to an odds ratio of approximately 0.084 (0.609^5^ ≈ 0.084), indicating that the odds of cognitive impairment would be reduced to about 8.4% of the baseline odds, equivalent to roughly a 91.6% reduction in odds. Similarly, a 5-point increase in ADL corresponds to an odds ratio of approximately 0.051 (0.553^5^ ≈ 0.051), meaning that the odds of cognitive impairment would be reduced to about 5.1% of the baseline odds (approximately a 94.9% reduction in odds). However, given the observational design and potential residual confounding, these estimates should be interpreted cautiously.

Whether improved functional capacity leads to better cognition, or whether preserved cognition facilitates independence, cannot be determined in this study. Diabetes mellitus was associated with higher odds of cognitive impairment. Chronic hyperglycemia and insulin resistance have been proposed to contribute to microvascular damage, oxidative stress, and inflammatory processes affecting hippocampal and prefrontal regions, mechanisms consistent with prior research in schizophrenia populations (21). However, the large odds ratio accompanied by a wide confidence interval indicates limited precision and potential model instability. Accordingly, this association should be interpreted cautiously and regarded as exploratory. Importantly, the predictive performance of the model was evaluated using the same dataset employed for model development. In the absence of internal cross-validation or external validation, performance metrics may be optimistic, and replicability cannot be ensured.

### 4.3 Cognitive and social function phenotype clustering and nursing implications

K-means clustering identified three data-driven cluster patterns: optimal functioning (Cluster 1), mild-to-moderate impairment (Cluster 0), and severe impairment (Cluster 2). The clustering results revealed distinct patterns across cognitive function, social support, and disease characteristics, which may provide preliminary insights for stratified clinical consideration. Cluster 2 patients, characterized by low cognitive function, insufficient social support, and prolonged disease duration, may require more intensive multidisciplinary support. Patients within this phenotype may benefit from intensified multidisciplinary interventions, including structured cognitive rehabilitation targeting memory, attention, and executive function; strengthened family and community engagement; optimization of chronic disease management (particularly glycemic control); and individualized functional rehabilitation programs. Such heterogeneity is consistent with previous research indicating substantial cognitive and functional variability within schizophrenia populations across the lifespan (22). Prior studies have also applied clustering or subtype approaches to identify distinct cognitive or biological profiles in psychosis, highlighting the value of phenotype-oriented stratification in clinical research (23).

Patients in Cluster 0 may benefit from early supportive strategies to maintain current functioning, while Cluster 1 patients may focus on sustaining stability and preventing relapse. This stratified approach aligns with international recommendations emphasizing individualized care based on functional status in elderly patients with schizophrenia (24). However, given the absence of external validation, stability testing, or independent replication, these phenotypes should be interpreted as exploratory patterns rather than definitive subtypes.

### 4.4 Guidance for future nursing practice

Based on the observed associations and phenotype patterns, this study suggests several potential directions for clinical nursing practice.

- Cognitive-focused strategies: structured cognitive rehabilitation programs may be prioritized for patients with evident impairment, particularly those in the severe phenotype, Cluster 2. Working memory impairment is widely recognized as a core cognitive deficit in schizophrenia and may significantly affect functional outcomes (25).
- Chronic disease management: given the observed association between diabetes and cognitive impairment, attention to metabolic control—including glucose monitoring, lifestyle guidance, and medication management—may be relevant components of comprehensive care.
- Enhancing social support and social functioning: Given the associative nature of the findings, psychosocial support may warrant consideration within comprehensive nursing frameworks. Interventions aimed at strengthening family involvement, psychological support, and community resource linkage may be considered in supportive care planning. Social skills training (SST), which has demonstrated efficacy in improving social functioning in individuals with schizophrenia (26), may serve as one structured approach to enhance interpersonal competence and perceived support.
- Promoting functional independence: individualized rehabilitation, life-skills training, and environmental adaptations may support daily functioning.
- Stratified multidisciplinary collaboration: nursing teams may collaborate with psychiatrists, rehabilitation therapists, nutritionists, psychologists, and social workers to provide integrated care tailored to phenotype characteristics. Cognitive behavioral therapy for psychosis (CBTp) may serve as a complementary approach for addressing persistent symptoms that influence cognitive and functional outcomes. The development and clinical application of CBTp were strongly influenced by the pioneering work of Kingdon and Turkington, who demonstrated its effectiveness in reducing psychotic symptoms and improving coping strategies in individuals with schizophrenia (27, 28).
- Personalized education: patient and caregiver education regarding cognitive impairment and chronic disease management may improve adherence and long-term stability.

Although causal relationships cannot be established in this study, the findings may provide a framework for developing hypothesis-driven and stratified nursing strategies in elderly patients with schizophrenia. Future studies incorporating medication-related and symptom-related variables are needed to better clarify these relationships.

## 5 Study significance and limitations

This study systematically evaluated cognitive function, social support, functional independence, and metabolic indicators in elderly patients with schizophrenia, reporting the high prevalence of cognitive impairment and identifying its main influencing factors. Cluster analysis further distinguished clinical phenotypes, providing a basis for stratified nursing management. The findings suggest that clinical nursing may benefit from integrating multidimensional approaches encompassing cognition, psychosocial support, and chronic disease management.

Several limitations should be acknowledged. First, it was a single-center retrospective study with a relatively limited sample size, which may restrict generalizability and contribute to statistical imprecision. The wide confidence interval observed for diabetes mellitus suggests possible instability, and findings should be interpreted cautiously. Second, the cross-sectional design precludes causal inference. Variables such as ADL and social support may represent consequences rather than independent determinants of cognitive impairment, and reverse causation cannot be excluded. Third, although the MoCA is widely used as a cognitive screening tool, applying the conventional cutoff (<26) in an elderly psychiatric population with relatively low educational attainment may have overestimated the prevalence of cognitive impairment, despite education adjustment. Future studies may consider education-adjusted normative data specific to elderly psychiatric populations. Fourth, important clinical and pharmacological variables, including antipsychotic type and dosage, anticholinergic burden, negative and depressive symptom severity, polypharmacy status, and treatment resistance, were not systematically assessed. Predictor pre-selection may also have inflated model performance. Finally, the clustering analysis was exploratory and lacked validation; therefore, the identified phenotypes should be regarded as hypothesis-generating. Future multicenter prospective studies with larger samples, comprehensive clinical assessments, and validation analyses are warranted to confirm and extend these findings.

## 6 Conclusion

Cognitive impairment is highly prevalent among elderly patients with schizophrenia. Greater perceived social support and functional independence were independently associated with lower odds of cognitive impairment, whereas diabetes mellitus was associated with higher odds. Phenotype identification via clustering analysis may provide a framework for stratified nursing management. Given the observational design, these findings should be interpreted as associative rather than causal. Prospective and interventional studies are warranted to further examine these relationships and evaluate targeted care strategies.

## Data Availability

The original contributions presented in the study are included in the article/**Supplementary Material**. Further inquiries can be directed to the corresponding author.
