# Supplementary figures and images for: Cognitive impairment and associated factors in elderly patients with schizophrenia: a retrospective observational study with phenotype analysis
Source: Front Psychiatry. 2026 Apr 30;17:1789211. doi: 10.3389/fpsyt.2026.1789211 (PMC13171557; doi:10.3389/fpsyt.2026.1789211)

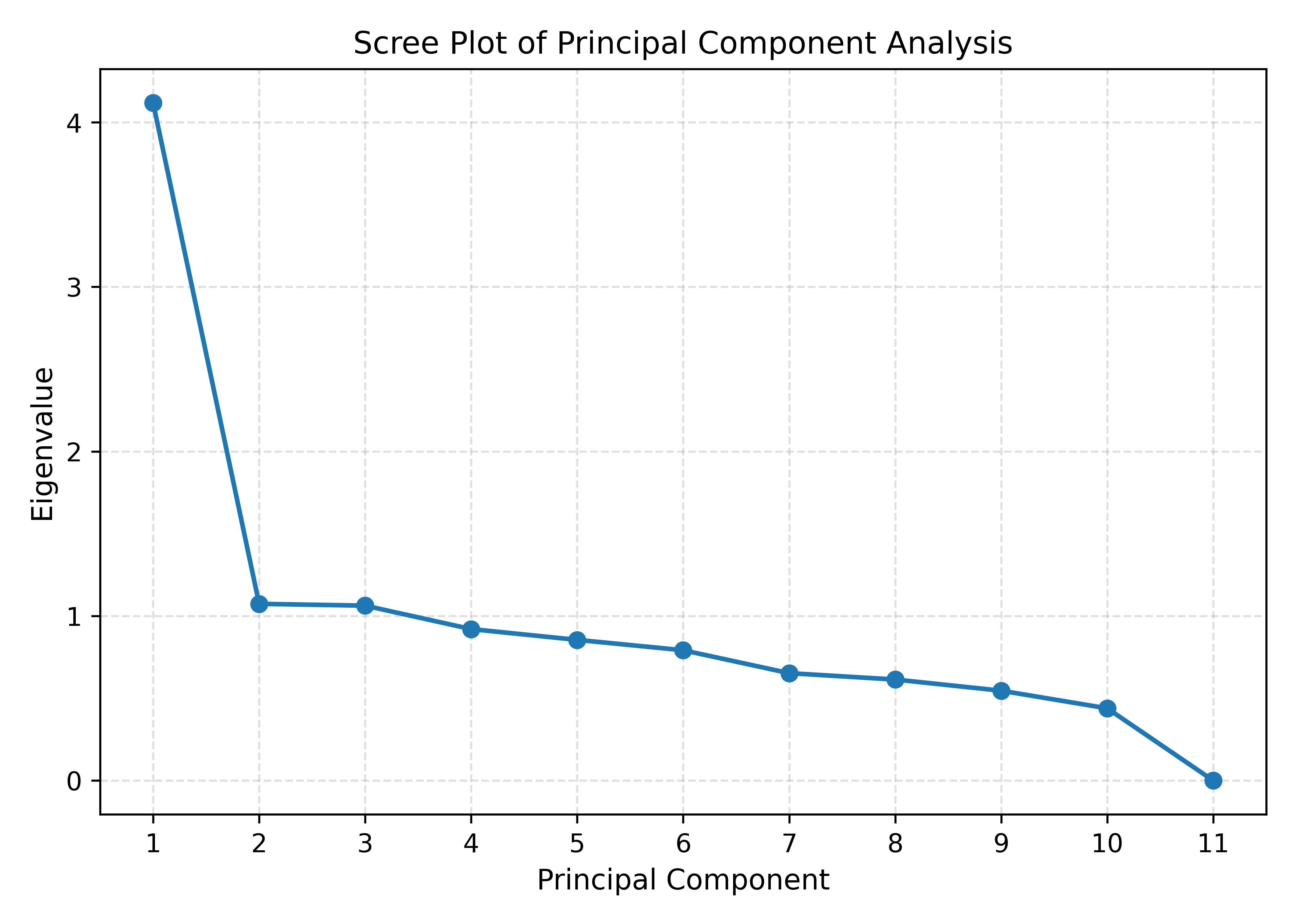

Supplement: Supplementary file 1 [file Image1.jpeg]
