# Supplementary material for: Cognitive impairment and associated factors in elderly patients with schizophrenia: a retrospective observational study with phenotype analysis
Source: Front Psychiatry. 2026 Apr 30;17:1789211. doi: 10.3389/fpsyt.2026.1789211 (PMC13171557; doi:10.3389/fpsyt.2026.1789211)
Supplement: Supplementary file 2 [file Table1.docx]

Supplementary Table S1. Factor loadings from PCA (unrotated solution).

| **Variable** | **PC1 loading** | **PC2 loading** |
| --- | --- | --- |
| MoCA | 0.78 | 0.12 |
| ADL | 0.73 | 0.25 |
| MSPSS total | 0.70 | 0.18 |
| Disease duration | -0.62 | 0.33 |
| Relapses | -0.58 | 0.41 |
| Education | 0.55 | -0.2 |
| BMI | 0.05 | 0.49 |
| Age | 0.11 | 0.52 |

Loadings ≥ |0.40| are highlighted in bold.
